# Supplementary figures and images for: Rift Valley fever virus targets the maternal-foetal interface in ovine and human placentas
Source: PLoS Negl Trop Dis. 2020 Jan 21;14(1):e0007898. doi: 10.1371/journal.pntd.0007898 (PMC6994196; doi:10.1371/journal.pntd.0007898)

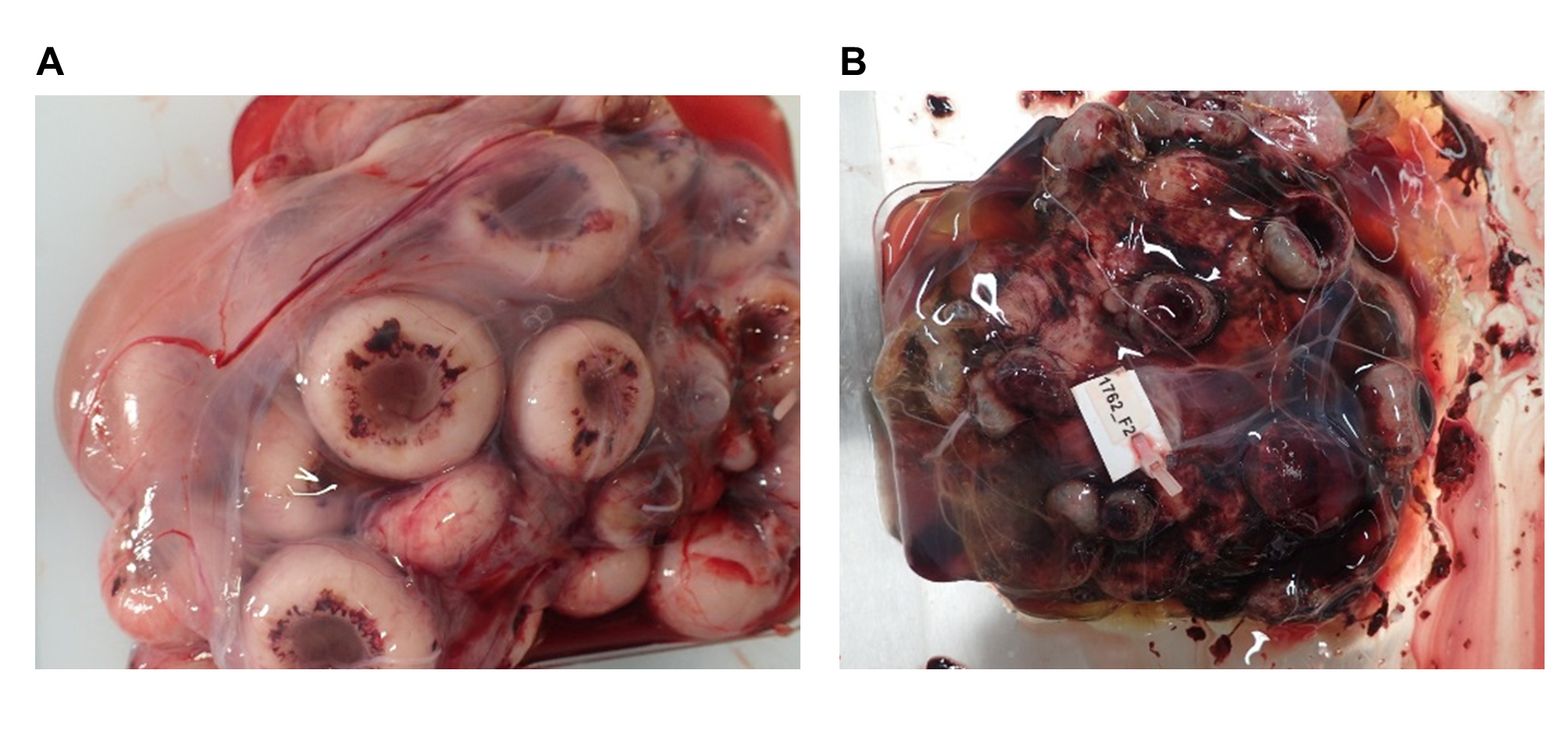

Supplement: S1 Fig — Placenta from a healthy ewe (A) and from an ewe inoculated with RVFV, necropsied six days after inoculation (B). Placentas were collected during experiment 1 at one third of gestation. (TIF) [file pntd.0007898.s001.tif]

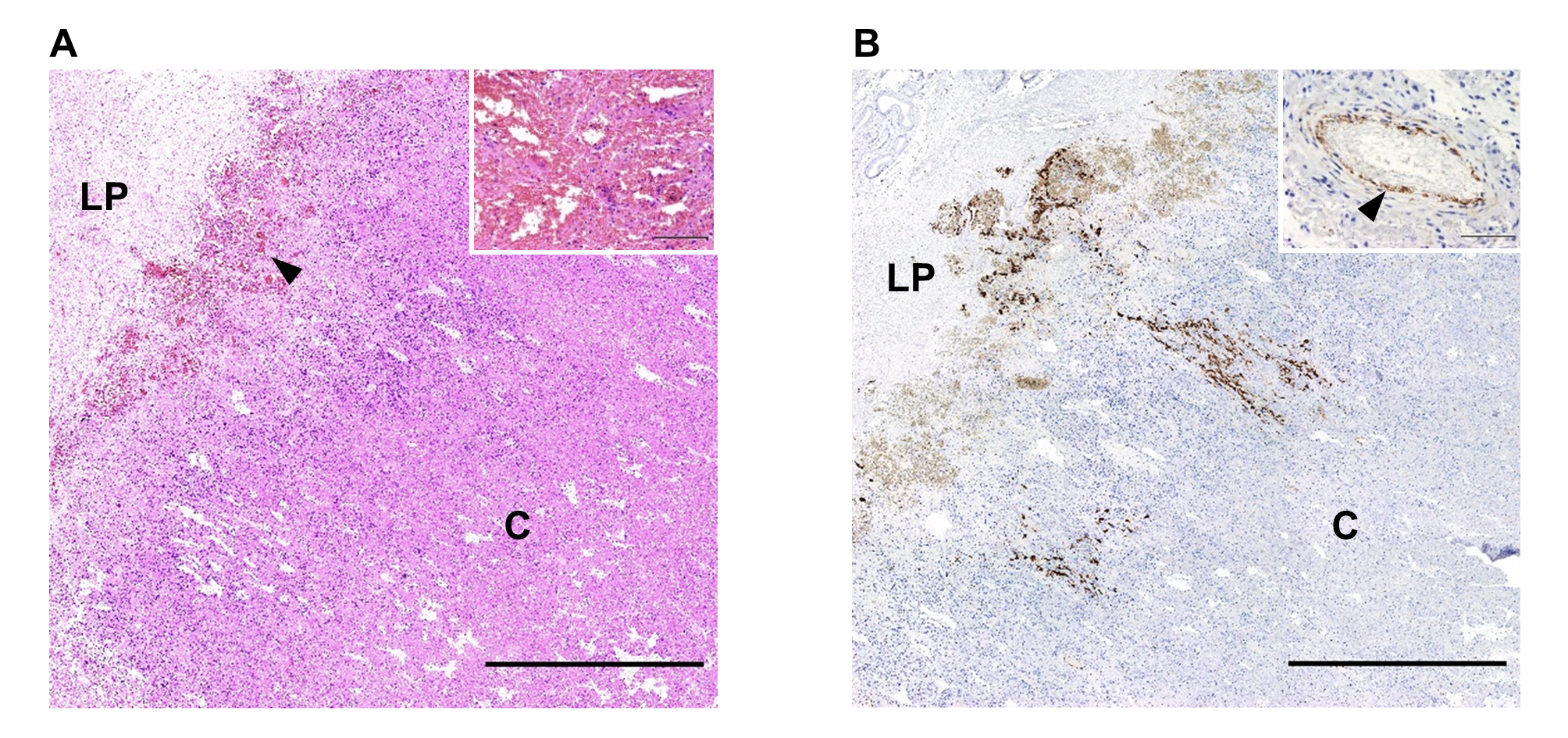

Supplement: S2 Fig — (A) Micrograph of HE-stained caruncle tissue of ewe 1845 euthanized at 7 dpi (experiment 2). Notice the extensive haemorrhages at the base of the caruncle (arrowhead and inset). (B) Immunohistochemical staining of RVFV antigen. Positive staining is only seen in those areas where necrotic maternal epithelium is still present. Some maternal blood vessels are also stained (arrowhead and inset) and show the presence of RVFV antigen in the endothelial cells and the smooth muscle cells of the tunica media (inset). Bar = 1000 μm (A, B), 100 μm (inset A), or 50 μm (inset B). LP; lamina propria, C; caruncle. (TIF) [file pntd.0007898.s002.tif]

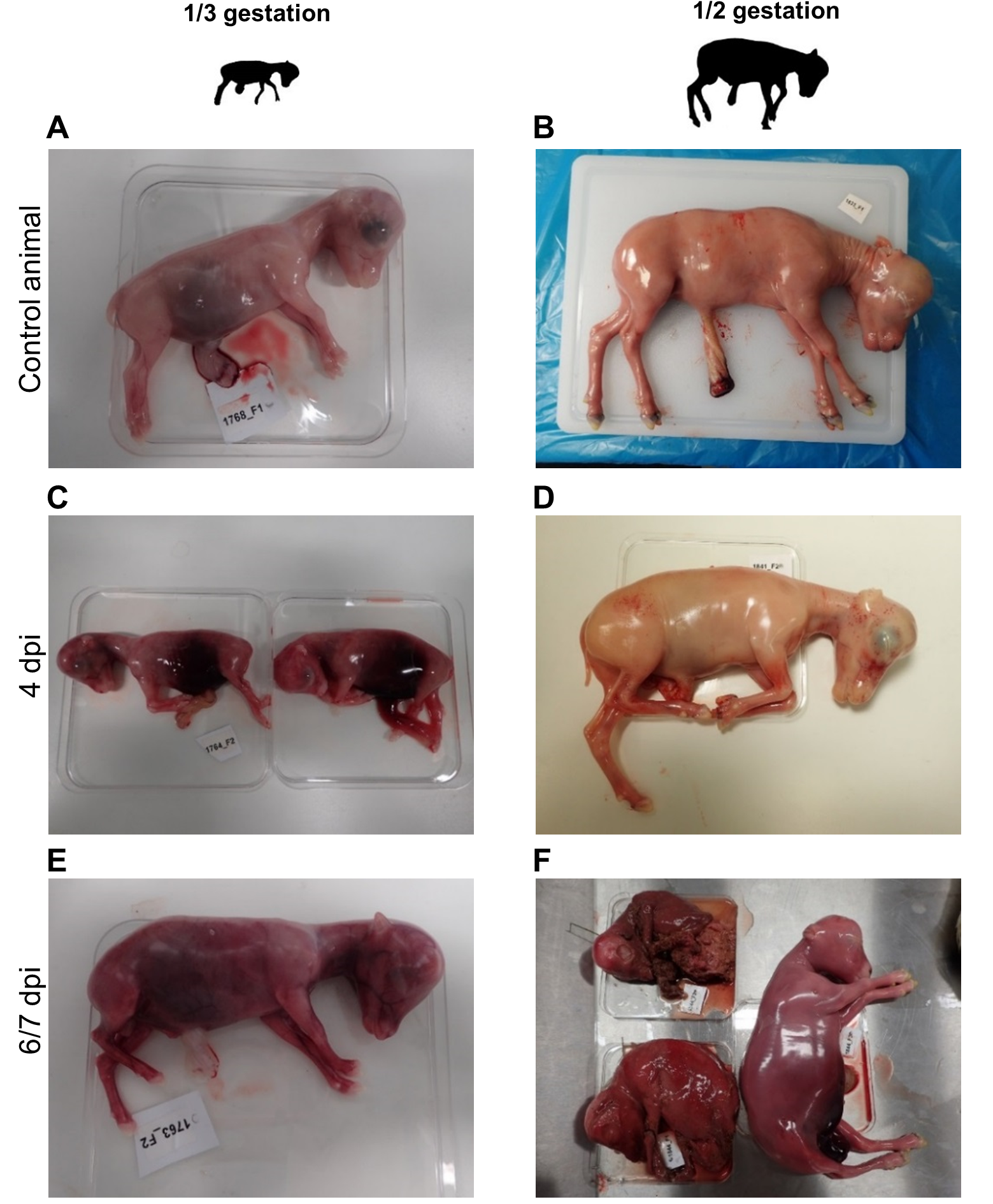

Supplement: S3 Fig — Healthy foetuses collected from ewes necropsied at one third (A) or at mid-gestation (B). (C) Foetuses carried by ewe 1764 that succumbed 4 days after inoculation with RVFV in experiment 1. (D) Live foetus collected from an ewe that was necropsied at 4 dpi in experiment 2. (E) Autolytic foetus collected from an ewe necropsied at 6 dpi in experiment 1. (F) Two aborted foetuses from experiment 2 (left) and one foetus (right) that was still inside the uterus at the moment of necropsy. (TIF) [file pntd.0007898.s003.tif]

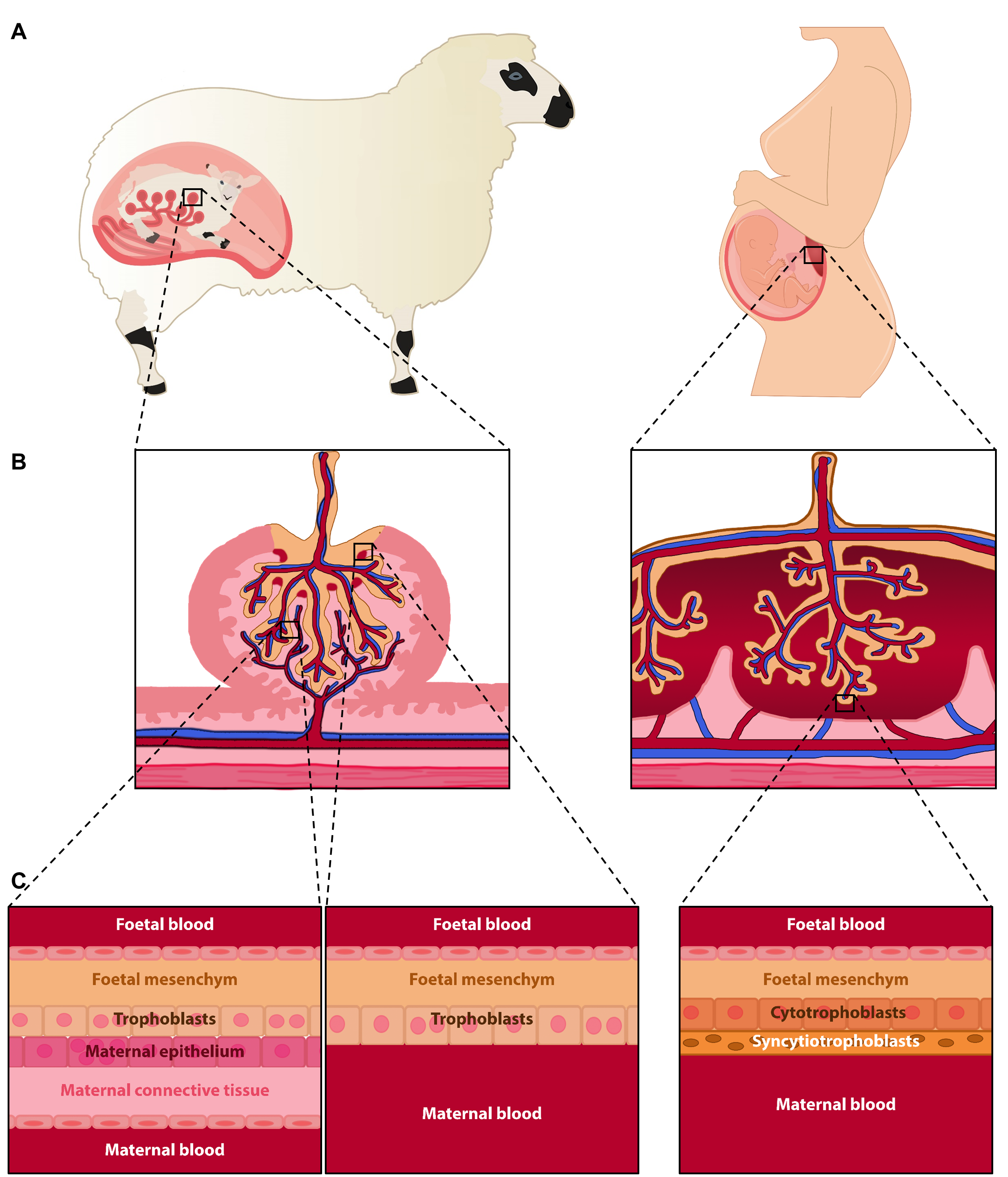

Supplement: S4 Fig — A human placenta consists of a single discoid plaque whereas an ovine placenta consists of placentomes (A). A cross section of both placentas is depicted, showing the maternal tissues in shades of pink, and the foetal villi in orange. Blood and arteries are depicted in red, veins are depicted in blue (B). In the synepitheliochorial placenta (C, left panel), the foetal blood is separated from maternal blood by several maternal and foetal cell layers. In the haemophagous zone (C, middle panel) maternal blood is in direct contact with the foetal trophoblasts, which is similar to the human haemochorial placenta (C, right panel). (TIF) [file pntd.0007898.s004.tif]
