# Supplementary material for: Rift Valley fever virus targets the maternal-foetal interface in ovine and human placentas
Source: PLoS Negl Trop Dis. 2020 Jan 21;14(1):e0007898. doi: 10.1371/journal.pntd.0007898 (PMC6994196; doi:10.1371/journal.pntd.0007898)
Supplement: Attachment — Submitted filename: Response to Reviewers.docx [file pntd.0007898.s005.docx]

**Response to reviewers**

**Manuscript “Rift Valley fever virus targets the maternal-foetal interface in ovine and human placentas” (#PNTD-D-19-01103)**

We thank both reviewers for their positive evaluation of our manuscript and their suggestions for improvement. Our responses to each of the comments and suggestions are given below. Line numbers refer to the revised manuscript. Please note that we changed the numbering of figures to comply to the instructions to authors and we have made some additional editorial changes, which are all marked in the “Revised Article with Changes Highlighted” file. Finally we have replaced reference 1 with a more appropriate reference and we have moved Figure 8 to the Supporting information section (now S4_Fig).

**Reviewer #1:** Rift Valley fever virus (RVFV) is an arbovirus of the order Bunyavirales that causes severe disease in ruminants and humans. Surprisingly, pathology resulting in abortion remains poorly described. In the present work, pregnant ewes were inoculated intravenously with a highly virulent strain of RVFV and necropsied at different time points post inoculation. Tissues were collected and analyzed by real-time qPCR, virus isolation, and immunohistochemistry. Results reveal that maternal epithelial cells are the first placental cells to be infected, followed by fetal trophoblasts. In addition, RVFV can bypass the maternal epithelial cell layer by directly targeting fetal trophoblasts in the hemophagous zone, a region of the ovine placenta where maternal blood is in direct contact with fetal cells. Abortion was associated with widespread necrosis of placental tissues and severe hemorrhage. Experiments with human placental explants revealed efficient infection and replication in both cyto- and syncytiotrophoblasts. Results demonstrate that RVFV targets the fetal-maternal interface in both ovine and human placentas. The virus crossed the ovine placental barrier via two distinct routes, ultimately resulting in placental and fetal demise followed by abortion. Finding show that the same RVFV strain replicates efficiently in human trophoblasts and underscores the risk of RVFV infection during human pregnancy.

This is an excellently executed and well-illustrated study. Authors have nicely analyzed and compared ovine and human placental tissue with respect to RVFV infection.

It remains unclear how closely human placental explants mimic the in situ situation. In order to infect an ovine fetus a pathogen has to overcome several tissue layers compared to human placental tissue. This could indicate that human fetal infection should occur very frequently.

Authors report ….. “In addition, RVFV can bypass the maternal epithelial cell layer by directly targeting fetal trophoblasts in the hemophagous zone, a region of the ovine placenta where maternal blood is in direct contact with fetal cells.” Does this represents a back-door for any kind of placental infection in ruminants?

**Response**: We much appreciate the kind words of this reviewer and this acknowledgement of our work. Regarding the question “*Does this represent a back-door for any kind of placental infection in ruminants*”? The haemophagous zone may indeed function as a “back-door” for arboviruses that are unable to replicate in maternal epithelial cells.

**Responses to specific comments:**

1. Conditions of RVFV PCR including used primers should be provided in more detail.

**Response**: We have described the PCR in more detail, including used primers, in the Materials and Methods section as requested (Lines 197-201).

2. Table 1 should be accompanied by a more informative legend explaining the different colours, NT, abbreviations, mentioned internal case numbers, meaning of 1/3 and ½ gestation etc. Table should be readable without reading the remaining manuscript.

**Response**: We agree with the reviewer and we have adapted the table and improved the caption.

3. Numbering of figures starting at line 253 should occur in numeric/alphabetic order (e.g. please start with Figure 1a and not 1e).

**Response**: Please note that Figure 1 describes both Experiment 1 and Experiment 2. The order is numeric, starting in Line 242.

4. It would be interesting to know whether alive foetuses of experiment 2 would develop long-term alterations in any other organ?

**Response**: We expect that foetuses necropsied at 4 or 7 dpi during experiment 2, although still alive at the moment of necropsy, would have succumbed to the infection had pregnancy been allowed to continue, as all tested placentomes were strongly positive for both viral RNA (PCR) and viral antigen (IHC). Detection of viral RNA in about half of the foetal liver samples with negative staining in IHC suggest that virus replication was at a relatively early stage when ewes and foetuses were necropsied. Continued virus replication in placental tissues and foetal livers (as well as other organs) would almost certainly have resulted in placental and foetal demise.

5. How would the authors explain the difference with respect to placental/foetal susceptibility between experiment 1 and 2?

**Response**: Although there are indeed some differences notable, we do not feel that these differences are sufficiently clear to make solid conclusions. One notable difference is that the foetuses collected on dpi 4 in experiment 1 were found dead, whereas the foetuses collected on dpi 4 in experiment 2 were all alive. However, it is important to note that the foetuses in experiment 1 were aborted. In our opinion, the results of both experiments suggest that foetuses are infected before day 4 and that abortion generally occurs around day 7.

6. Line 272: it is stated: “…..observed an increase in mass of the foetal and maternal villi with concomitant decrease in mesenchyme (meaning mesenchymal tissue?)…….”. How was this statement substantiated (e.g. quantitative or semi-quantitative analysis?)? Furthermore, how would authors explain this tissue enlargement and decrease in mesenchymal tissue within such a short time period, especially in experiment 1? What about placental tissue of experiment 2 without macroscopic lesions?

**Response**: The increase in mass of placentomes during gestation is well-described. As pregnancy progresses, the exchange of nutrients and waste products between mother and foetus has to intensify. To make this possible, the maternal and foetal villi increase in mass replacing foetal mesenchyme. The differences that the reviewer refers to are not due to virus infection (the H&E photos are of placentomes from control animals), but due to the difference in gestation of the animals.

7. The question remains whether the extensive hemorrhage in placental tissue is solely due to a direct virus effect or represents a secondary event, at least in part, mediated by (maternal?) liver damage.

**Response**: Indeed, maternal liver damage probably contributed to the severity of the haemorrhages observed in the placenta as the liver is responsible for the production of coagulation factors. In other words, liver damage is likely to predispose for bleeding. This was addressed in the Discussion section (Lines 378-380).

8. Overall, I miss a comment on the pathology found in the ewes. It would be important to know whether animals suffered from severe liver damage, which could complicate or contribute to placental lesions. Furthermore, is there a preferential infection of placentomes during pregnancy or did animals suffer predominantly from liver necrosis and placental lesions represent a concurrent but less severe organ manifestation?

**Response**: We thank the reviewer for this important comment. In all ewes, signs of multifocal necrotizing hepatitis were noted (Lines 250-251), which indeed could contribute indirectly to placental lesions (see also response to comment #7). Regarding the second question, our results confirm that the liver is a primary target organ of RVFV, but for the first time show that the virus replicates to even higher levels in the placenta. We also show that virus replication continues in the placenta after the acute phase (after replication in the liver and viremia have waned). We also would like to note that the lesions in placentae (if pregnancies are allowed to continue) are very severe. Whereas the liver can regenerate when animals survive the infection, there are not indications that the placenta can recover from RVFV infection.

9. It remains unclear whether abortion is due to placental necrosis, drop in systemic progesterone levels or systemic effects of RVFV infection in the ewe.

**Response**: We agree with the reviewer that more research is needed to firmly establish the pathogenic events that result in abortion. However, based on our findings, it is plausible to propose that the following events are involved:

1. Lowered coagulation factors predispose for bleeding;
2. Necrosis of maternal epithelium of the placenta results from virus replication. This results in lowered exchange of nutrients and waste products between mother and foetus;
3. Placental demise results in lowered progesterone levels;
4. Foetuses are infected, resulting in foetal liver necrosis and death (note that in some cases the foetus is expelled before being reached by the virus).
10. I miss a comment on the vasculature in the affected placentas (e.g. necrosis, vasculitis?). It is stated that…”blood vessels were found that stained positively for RVFV in the endothelium and/or smooth muscle cells in the blood vessel wall…”.in line 303/304. In addition, the prominent endothelial staining in figure 6 raises the question whether RVFV infection triggered morphologically recognizable or functional cell damage. Did these vessels also display necrosis (caspase 3, TUNEL-positivity?)?

**Response**: Thank you for pointing this out. We have not observed any sign of pathology in blood vessels and have added a note on this in the results section (Lines 317-318).

11. Line 320: what is meant by …endothelial staining…(e.g. immunohistochemistry for endothelial cells or RVFV?)?

**Response**: We apologize for not stating clearly that RVFV antigen was detected in endothelial cells. We have modified the sentence accordingly (Lines 335-336).

Minor points:

12. Figure 4: Please indicate (mark) more clearly the fetal and maternal part of the placentome.

**Response**: We have modified this figure as requested.

13. Findings in figure 7 should be depicted at higher magnification for c and d, in addition.

**Response**: We have included higher magnifications, but we have also included a staining for cytokeratin to more clearly identify the epithelial cells (cytotrophoblasts and syncytiotrophoblasts).

14. Blood-tingled number tags should be removed in the supplementary file figure 1 and 2.

**Response**: Unfortunately, blood-tinged tags cannot be removed without manipulating the photographs and we do not have pictures that are completely free of blood stains. We hope that this is acceptable but will take this into account in future submissions.

15. Supplementary file figure 1 and 2: Please provide a more explanatory, descriptive legend.

**Response**: We agree with the reviewer and have expanded the legends as requested.

16. Supplementary file figure 3b: Positive maternal vessels are not recognizable at the tip of the arrow.

**Response**: We agree with the reviewer. The maternal vessels are now indicated in the inset.

17. Mesenchyme is spelled differently in figure 3 and the text (line 273 and 374).

**Response**: Thank you for pointing out this mistake, which is now corrected.

**Reviewer #2:** Livestock are highly susceptible to infection with Rift Valley fever virus (RVFV). Pregnant ewes suffer fetal abortions when infected in nature, and this massive fetal infection has been known since the time that RVF was first described. However, the mechanism by which RVFV infects the ovine placental has not been thoroughly documented in either a natural or experimental setting, and this manuscript addresses this important gap in the field. The authors perform experimental inoculation of pregnant sheep at 2 time points during gestation. The ewes and their fetuses are monitored for RVFV infection and pathology. The authors are to be commended for completion of a difficult study. Given the limitations of a large animal study with a high-containment agricultural pathogen, the data presented here contribute significantly to our understanding of livestock placental infection by RVFV. Comments are mostly minor for clarity of text and data presentation. This reviewer would caution the authors to avoid unnecessary speculation in the discussion. While the histology was limited to H&E and basic IHC, this reviewer feels the data largely support the conclusions.

Specific comments:

1. Line 54 – Please clarify that the severe outcome of RVFV during LIVESTOCK pregnancy is well documented. The effect in human pregnancy is not as clear.

**Response**: The reviewer is correct, this was adapted (Line 53).

2. Paragraph beginning at line 89 and ending at 105 describing the ovine placental structure should reference the appropriate citations.

**Response**: We have added an appropriate reference (Line 93): F.B.P. Wooding and G.J. Burton. Synepitheliochorial placentation: ruminants (ewe and cow). *In* : Comparative placentation. Structures, Functions and Evolution. 2008 Springer-Verlag, Berlin Heidelberg, Germany.

3. Line 143-44: Please clarify what “right upfront the national breeding season” means.

**Response**: This has been removed from the text as it is irrelevant to the study and may cause confusion. The sheep were treated with progesterone when the mating season started.

4. Line 171: Please clarify what 1% a/a means.

**Response**: This means antibiotic/antimycotic, which is explained before in the text (Line 119).

5. Throughout the manuscript, the term “virus isolation” is used when it appears to refer to infectious virus quantification by TCID50.

**Response**: The term ‘virus isolation’ is used throughout the manuscript when infectious virus has been isolated from an organ or plasma, which is measured by quantifying infectious virus in an end-point dilution assay.

6. Results section beginning on line 235: Please provide information on how the inoculum dose and the gestation time points were chosen.

**Response**: This was added to the Results section (Lines 236-242).

7. Line 257: “dpi 7” should be “7 dpi”

**Response**: Corrected.

8. Line 275-277: The authors refer to hemorrhage of the uterine wall and placentomes of ewe 1764– does this refer to macroscopic hemorrhage (as seen in Supp Fig 1) or microscopic hemorrhage visible by histology?

**Response**: This refers to macroscopic haemorrhages, which has been clarified in the text in (Line 287-288).

9. Line 307 – remove the comma after Experiment 1.

**Response**: Done as requested.

10. Line 313-315 – Do the authors feel that the 4/8 dead fetuses without vRNA in the liver were not directly infected but rather died due to destruction of the associated placenta?

**Response**: Yes, indeed, this is noted in the Discussion section (Lines 360-361).

11. Lines 348-365 – Description of sheep pregnancy development needs the addition of references.

**Response**: We have added this reference, see comment 2.

12. Line 356-365 – Since neither progesterone nor cytokine levels were measured in this study, the conclusions from this paragraph are speculative.

**Response**: Indeed, but we believe it is nevertheless valuable to make these propositions in the Discussion section.

13. Lines 366-376 – Description of two routes that RVFV uses to cross the placenta is also largely speculative. The data in Fig 4 clearly show a lot of viral antigen within the placenta at 6 dpi, but without a true serial sacrifice experiment, it is difficult to definitively say what ‘route’ is used.

**Response**: We appreciate this comment and although we agree that a more extensive serial sacrifice experiment would be valuable, we do believe that the present data convincingly show that (at least) two routes are used by the virus to cross the placenta: one preceded by replication in maternal epithelial cells and one via the haemophagous zone.

Comments regarding figures:

14. Fig. 1 – please show rectal temperatures of the control group

**Response:** Rectal temperatures were added and we have also added PCR data from the control group.

15. Fig. 4 – What counterstain is used for the IHC staining? How certain are you of the trophoblast cell identification without cell-specific markers or a counterstain? It would be helpful to have a square or box in (a) and (d) indicating the zoomed-in areas shown in b, c, e, and f. It would also be helpful to have uninfected control placentomes stained for IHC visualize any background. In addition, labeling of the structures in Fig 4 (similar to the labeling in Fig 3c,d) would help to orient the reader since most readers are not intimately familiar with placental histology. There does not seem to be an arrow in 4b that is referred to in the legend.

**Response**:

-Counterstaining was performed with haematoxylin. This is now added to the M&M (Line 231-232).

-We have added zoomed-in pictures as insets.

-We have added pictures of uninfected control placentomes (Fig. 4D, E, I, J).

-An arrowhead has been added.

16. Fig. 5 – There is no black asterisk that is referred to in the legend.

**Response**: We apologize for this. This is now added. We have also modified the colours of the lettering to make it more readable.

17. Fig. 7 – How many human placental donors were tested? How many tissue explants were tested per donor per time point? Is the data in this figure a compilation of replication from different donors or a single donor? What is the counterstain for the IHC? In c and d, are these tissue explants the cross section of a single villus? Please clarify what it is that the reader is looking at. In such a small piece of tissue, how confident are you of the cytotrophoblasts and syncytiotrophoblasts without a specific cell marker? Addition of arrows to indicate the different cell types would be helpful. Is there any background IHC staining in uninfected human placental explants? Control staining images would be helpful.

**Response**:

-Two human placentas were tested from different donors. Per time point 4 explants were tested. This figure is a compilation of results obtained from a single donor, however the results with the second donor were highly similar.

-Hematoxylin was used for counterstaining (see line 231-232).

-Fig. 7C and D (E and F in the revised manuscript) indeed show a cross section of a single villus. We now explain this in the caption. A placental villus contains a single layer of syncytiotrophoblasts and due to the many nuclei, this cell type is easily recognisable under a microscope. The cytotrophoblast layer is the epithelial layer under the syncytiotrophoblasts. At the end of pregnancy this layer disappears, however some cytotrophoblasts may still be present later in pregnancy.

-We have expanded the figure with a picture of an uninfected placental villus, showing the absence of background staining in IHC and we have included a picture of a sample stained with cytokeratin antibody. Cytokeratin staining is used to identify epithelial cells, but does not differentiate between cytotrophoblasts and syncytiotrophoblasts. However, these two cell types can be distinguished easily as the former contain a single nucleus whereas the latter are syncytial cells, containing many nuclei.

18. Supplemental Fig 1 – are the negative control placenta and the RVFV-infected placenta from the same day of gestation?

**Response**: Yes, both placentas were collected from ewes at mid gestation. This is now added to the caption.

19. Supplemental Fig 2 – same question for the control animals. Are they the same gestational age as the fetuses in c, d or e, f?

**Response**: The foetuses depicted in panels A, C and E were of the same gestational age (1/2 gestation) and the foetuses depicted in panels B, D and F were also of the same gestational age (1/3 or gestation) This is made clear at the top of the figure.
